# Effect of oral administration of *Kudoa septempunctata* genotype ST3 in adult BALB/c mice

**DOI:** 10.1051/parasite/2015035

**Published:** 2015-12-02

**Authors:** Meejung Ahn, Hochoon Woo, Bongjo Kang, Yeounghwan Jang, Taekyun Shin

**Affiliations:** 1 School of Medicine, Jeju National University Jeju 63243 Republic of Korea; 2 College of Veterinary Medicine, Jeju National University Jeju 63243 Republic of Korea; 3 Ocean and Fisheries Research Institute, Jeju Special Self-Governing Province, Pyoseon-myeon, Segwipo-si Jeju 63629 Republic of Korea

**Keywords:** *Kudoa septempunctata*, ST3 genotype, Foodborne disease, Myxozoa, Olive flounder, *Paralichthys olivaceus*

## Abstract

*Kudoa septempunctata* (Myxozoa: Multivalvulida) infects the muscles of olive flounder (*Paralichthys olivaceus*, Paralichthyidae) in the form of spores. To investigate the effect of *K. septempunctata* spores in mammals, adult BALB/c mice were fed with spores of *K. septempunctata* genotype ST3 (1.35 × 10^5^ to 1.35 × 10^8^ spores/mouse). After ingestion of spores, the mice remained clinically normal during the 24-h observation period. No spores were found in any tissue examined by histopathological screening. Quantitative PCR screening of the *K. septempunctata* 18S rDNA gene revealed that the *K. septempunctata* spores were detected only in the stool samples from the spore-fed groups. Collectively, these findings suggest that *K. septempunctata* spores are excreted in faeces and do not affect the gastrointestinal tract of adult mice.

## Abstract (fr)

*Kudoa septempunctata* (Myxozoa: Multivalvulida) infecte les muscles du cardeau hirame (*Paralichthys olivaceus*, Paralichthyidae) sous la forme de spores. Pour étudier l’effet de l’ingestion de spores de *K. septempunctata* chez les mammifères, des souris BALB/c adultes ont été nourries avec des spores de génotype ST3 (1.35 × 10^5^ à 1.35 × 10^8^ spores/souris). Après ingestion de spores, les souris sont restées cliniquement normales pendant la période d’observation de 24 heures. Aucune spore n’a été trouvée dans les tissus examinés par dépistage histopathologique. Le criblage par PCR quantitative du gène d’ADNr 18S de *K. septempunctata* a révélé que les spores étaient détectées seulement dans les échantillons de selles des souris qui ont ingéré des spores. Collectivement, ces résultats suggèrent que les spores de *K. septempunctata* sont excrétées dans les fèces et n’affectent pas le tube digestif des souris adultes.

## Introduction

The WHO has cautioned that raw fish consumption, which is increasing globally, is a risk factor for infectious diseases caused by foodborne microbes and parasites [11]. Infection with species of the genus *Kudoa*, a myxosporean parasite, causes post-mortem myoliquefaction, or so called “jelly meat”, in fish. The resulting impairment of fillet quality, which renders the product unmarketable, has severe consequences for the marine fisheries and aquaculture industries [4].

*Kudoa septempunctata* Matsukane, Sato, Tanaka, Kamata & Sugita-Konishi, 2010 [7], in the form of spores, parasitises the muscles of olive flounder, an important food fish in Asian countries [7, 10]. Since *K. septempunctata* infection is unapparent in olive flounder, sliced raw fish has inadvertently reached consumers. *Kudoa septempunctata* is a newly described myxosporean species of Multivalvulida that was first identified in 2010 in the trunk muscle of cultured olive flounder (*Paralichthys olivaceus*) imported from Korea to Japan [7]. *Kudoa septempunctata* spores are composed of six or seven shell valves and polar capsules [7], which are genetically classified into three groups: ST1, ST2 and ST3 [10]. It has been reported that *K. septempunctata* isolated from culture-raised and wild-caught flounder fish in Japan were of the ST1 or ST2 genotype, while those transported from Korea to Japan were of the ST3 genotype [10].

A previous study reported that the diarrhoegenic activity of *K. septempunctata* spores was dose-dependent in suckling mice [5]. Spores of *K. septempunctata* have been isolated from meal samples of raw olive flounder taken from patients with diarrhoea at 2–20 h after ingestion; such patients usually recover within 24 h, suggesting that *K. septempunctata* spores are associated with transient clinical symptoms (i.e., diarrhoea and emesis) [5]. Although previous studies were conducted to examine the pathogenicity of *K. septempunctata* spores in suckling mice [5], the effect of *Kudoa* spores on adult mice remains unsolved. The aim of this study was to investigate the effect of *K. septempunctata* ST3 spores on adult BALB/c mice.

## Materials and methods

### Spore preparation

*Kudoa septempunctata*-infected fish were periodically screened by the microscopic examination of crude suspensions of muscle at 400× magnification. Heavy infections were defined as >10^5^ spores/g of tissue. Fish found to be heavily infected were filleted for spore purification and infected muscle samples were fixed in formalin for haematoxylin and eosin staining.

About 2 g of infected olive flounder muscle tissue were sieved through a 200 μm nylon sheet after adding 10 mL of phosphate-buffered saline (PBS) (Life Technologies, Grand Island, NY, USA). The resulting extract was passed through 100 and 50 μm filters to remove debris and centrifuged at 1500× *g* for 15 min at 4 °C. The pellet, which potentially contained *K. septempunctata* spores, was suspended in 1 mL of PBS; the spores were counted using a haemocytometer, then further purified using Percoll density gradient centrifugation as described elsewhere [1]. The viability of the purified spores was assessed by the trypan blue exclusion test [9].

### Animal model

BALB/c mice (Orient Bio Inc., Seongnam, Korea) were bred in the Laboratory Animal Facility at Jeju National University (Jeju, Korea). All animal experiments were conducted in accordance with the Jeju National University Guide for the Care and Use of Laboratory Animals (Permit number: 2015-0013).

### Experimental design

Male 6-week-old mice (18–20 g each, *n* = 76) were used in this experiment. In a preliminary experiment, 6-week-old male BALB/c mice were divided into five groups (*n* = 40) (8 mice/cage). The four experimental groups received 0.1 mL of a *K. septempunctata* spore suspension in different concentrations (1.35 × 10^5^, 1.35 × 10^6^, 1.35 × 10^7^ and 1.35 × 10^8^ spores, respectively), while the control group received 0.1 mL of PBS intragastrically. The mice were monitored for watery stools in each cage during the 24-h observation period. No sign of watery diarrhoea was visualised in any group, prompting us to use million (10^6^) to ten millions (10^7^) of spores in the next experiment.

To determine the fate of *K. septempunctata* spores in mice, two groups (*n* = 24) were orally administered 0.1 mL (1.35 × 10^6^ and 1.35 × 10^7^ spores, respectively) of a *K. septempunctata* spore suspension, while the control group (*n* = 12) received 0.1 mL of PBS intragastrically. Experimentally fed mice were euthanised by cervical dislocation (in accordance with the Jeju National University Guide for the Care and Use of Laboratory Animals) at 0, 1, 3, 6, 12 and 24 h post-ingestion (hpi) (*n* = 2 per group for each interval). The various organs, including the gastrointestinal tract (stomach, small intestine, large intestine and rectum), kidneys, spleen and liver, were dissected and subjected to PCR (polymerase chain reaction) and histopathological analyses. Stool samples were collected from all groups, dissolved in PBS and filtered through a 50 μm nylon sheet to remove debris. The filtrates were stored in a deep freezer (−80 °C) prior to quantitative PCR (qPCR) screening.

### Histological studies

For histopathological analysis, the various organs including intestines of mice were fixed in 10% neutral buffered formalin, and processed for paraffin embedding. The paraffin-embedded tissues were cut at a thickness of 5 μm using a rotary microtome (Leica, Nussloch, Germany). The tissue sections were routinely stained with haematoxylin and eosin for the routine histopathological examination.

### PCR detection of *K. septempunctata*

Quantitative real-time PCR was used to detect *K. septempunctata* 18S ribosomal DNA [5]. DNA was extracted from various organs and the stool suspension filtrates using a QIAamp DNA Mini Kit (Qiagen, Venlo, Netherlands), following the manufacturer’s protocol. qPCR was performed using TaqMan Universal Master Mix (Applied Biosystems, Carlsbad, CA, USA). The primers and probes described by Kawai et al. [5] were used. All reactions were carried out using the StepOne™ system (Applied Biosystems) with an initial denaturation step (10 min, 95 °C) and 50 cycles of amplification consisting of 95 °C for 15 s and 60 °C for 60 s. The gene copy number was determined using the standard curve method with plasmid DNA containing a copy of the target gene as a control.

Conventional PCR primers were designed to detect two *K. septempunctata* mitochondrial genes: cytochrome *c* oxidase subunit I (*cox1*) and large subunit rRNA (*rnl*) [10]. Amplification was performed on a Mycycler thermocycler (Bio-Rad, Hercules, CA, USA) with Diastar™ Taq DNA polymerase (SolGent Co. Ltd., Daejeon, Korea) using the protocol of Takeuchi et al. [10]. Negative controls (without template DNA) were included to check for contamination. The PCR products were sequenced on an ABI 3730XL DNA analyser. Mitochondrial genes were subjected to a multiple sequence alignment using ClustalW (http://www.clustal.org) with MEGA v. 5.1. New sequences were deposited in GenBank as KU163620 and KU163621.

## Results and discussion

**Figure 1.**
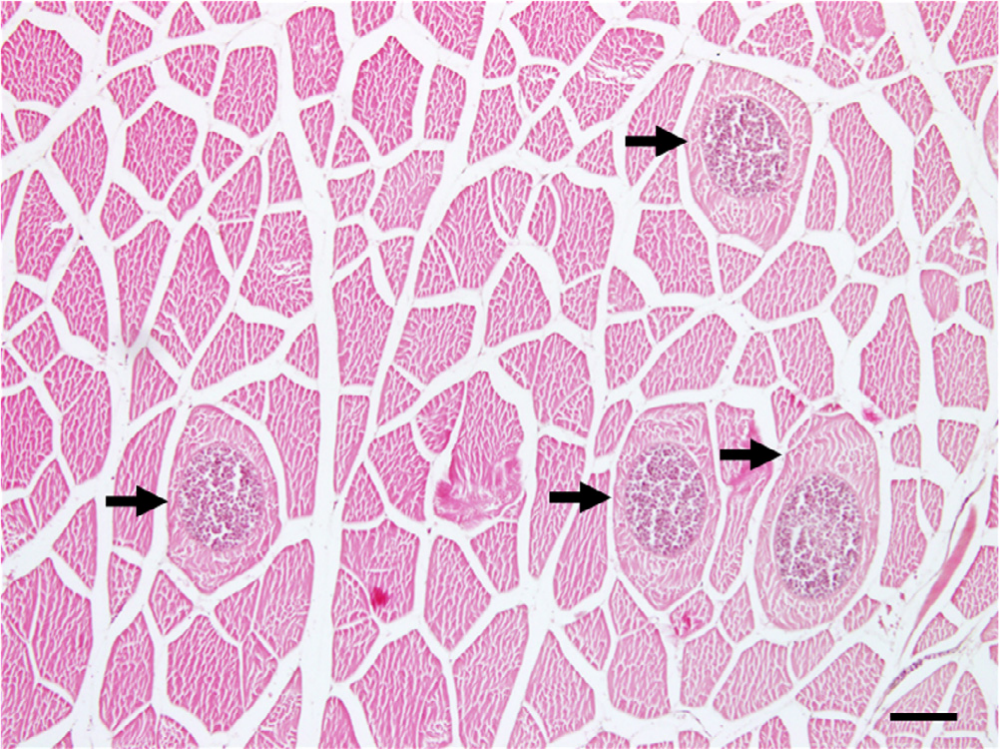
A histological section of olive flounder (*Paralichthys olivaceus*) muscles showing muscle fibres containing *Kudoa* spores. Arrows indicate spore-containing cysts. Haematoxylin and eosin-staining. Scale bar = 100 μm.

**Figure 2.**
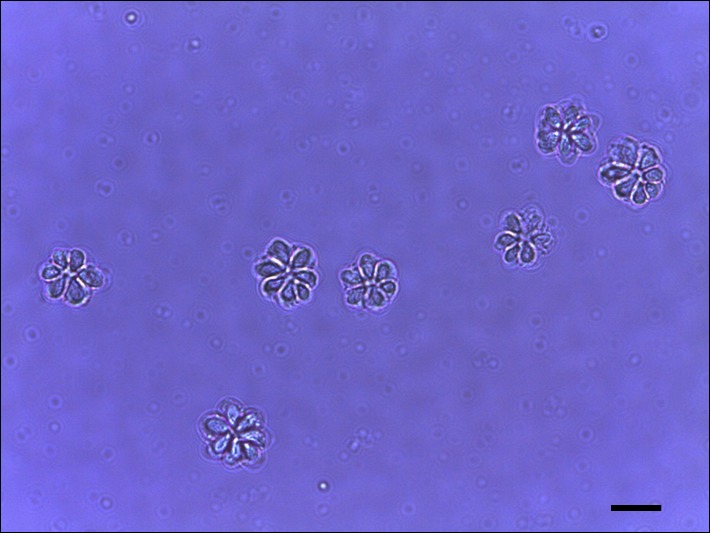
Phase-contrast micrograph of *Kudoa septempunctata* spores. Scale bar = 5 μm.

Histopathological examination of olive flounder muscle tissues collected from a Jeju fish farm occasionally showed the cysts of *Kudoa* spores in the muscles (Fig. 1, arrows). Isolated *Kudoa* spores were shown to contain six to seven shell valves and polar capsules per spore (Fig. 2). Sequence analysis of the mitochondrial genes *cox* and *rnl* revealed that the isolated *K. septempunctata* belonged to the ST3 genotype [10], and that it harboured *cox1-3* and *rnl-2* (GenBank accession numbers KU163620 and KU163621, respectively).

The administration to adult mice of various doses of *K. septempunctata* spores (1.35 × 10^5^ to 1.35 × 10^8^ spores/mouse) resulted in no significant diarrhoea in the present study. Table 1 shows the copy numbers of the *K. septempunctata* 18S rDNA gene in various organ and stool samples of mice that ingested *Kudoa* spores at 0, 1, 3, 6, 12 and 24 hpi. The *K. septempunctata* 18S rDNA gene signal was detected exclusively in the stool samples of experimental groups only after 3 hpi; in contrast, no signal was detected in any organs, including the whole intestinal segment (Table 1).

**Table 1.** *Kudoa septempunctata* 18S ribosomal DNA gene copy numbers in the various organs and stools of mice intragastrically administered with *K. septempunctata* spores.

Inoculated spores/mice	Samples	*K. septempunctata* 18S ribosomal DNA gene copy number (plasmid copies/4 μL)					
		0 hpi^a^	1 hpi	3 hpi	6 hpi	12 hpi	24 hpi
1.35 × 10^6^	Stomach	ND^b^	ND	ND	ND	ND	ND
	Small intestine	ND	ND	ND	ND	ND	ND
	Large intestine	ND	ND	ND	ND	ND	ND
	Rectum	ND	ND	ND	ND	ND	ND
	Stool	ND	ND	0.3	5.5 × 10^2^	1.6 × 10^1^	ND
	Kidney	ND	ND	ND	ND	ND	ND
	Spleen	ND	ND	ND	ND	ND	ND
	Liver	ND	ND	ND	ND	ND	ND
1.35 × 10^7^	Stomach	ND	ND	ND	ND	ND	ND
	Small intestine	ND	ND	ND	ND	ND	ND
	Large intestine	ND	ND	ND	ND	ND	ND
	Rectum	ND	ND	ND	ND	ND	ND
	Stool	ND	ND	0.4	1.2 × 10^3^	2.9 × 10^1^	ND
	Kidney	ND	ND	ND	ND	ND	ND
	Spleen	ND	ND	ND	ND	ND	ND
	Liver	ND	ND	ND	ND	ND	ND
PBS	Stomach	ND	ND	ND	ND	ND	ND
	Small intestine	ND	ND	ND	ND	ND	ND
	Large intestine	ND	ND	ND	ND	ND	ND
	Rectum	ND	ND	ND	ND	ND	ND
	Stool	ND	ND	ND	ND	ND	ND
	Kidney	ND	ND	ND	ND	ND	ND
	Spleen	ND	ND	ND	ND	ND	ND
	Liver	ND	ND	ND	ND	ND	ND

a hpi = hours post ingestion.

b ND = not detected.

**Figure 3.**
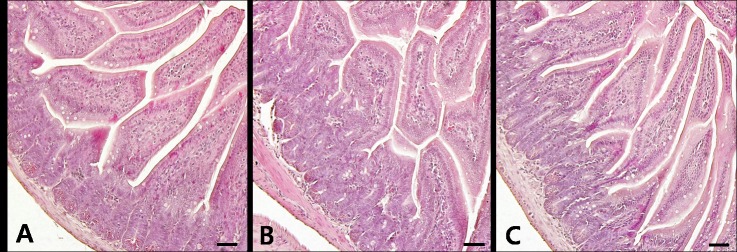
Representative photos of the small intestines of adult mice fed with vehicle (A) and *Kudoa septempunctata* spores (1.35 × 10^7^ spores/mouse) sacrificed at 6 h (B) and 12 h post ingestion (C). Histological findings of the small intestines of all experimental mice revealed no pathological changes in the intestinal villi as well as in the lamina propria of intestines. A–C: Haematoxylin and eosin-staining. Scale bars = 50 μm.

The gastrointestinal tract, devoid of faecal material, was carefully collected from mice in all of the groups and subjected to histological examination. No pathological changes of the intestines of vehicle-fed control mice were noted (Fig. 3A). Histopathological screening of the small intestines of Kudoa spore-fed mice revealed that Kudoa spores neither disrupted intestinal structures nor induced inflammation in mice fed with either 1.35 × 10^6^ spores or 1.35 × 10^7^ spores of *K. septempunctata* at 6 hpi and 12 hpi (Figs. 3B and 3C). These findings are also matched with those of PCR results (Table 1).

As far as Kudoa spores in suckling mice are concerned, Kawai et al. [5] reported that *K. septempunctata* spores caused diarrhoea with watery stools following a short latent period in the suckling-mouse model. In Kawai et al.’s study [5], neither monitoring for invasion of the intestines by *K. septempunctata* spores in suckling mice nor testing of adult mice was performed. In contrast, a recent paper reported that oral administration of Myxozoan spores (*Myxobolus honghuensis* spores) to suckling mice caused no pathological symptoms including diarrhoea and elevated fluid accumulation [2]. In our supplementary experiment of the pathogenicity of *K. septempunctata* spores in suckling ddY mice, no pathological symptoms (e.g., diarrhoea) were found (data not shown), in accordance with results obtained using other Myxozoan spores [2]. Considering that outbreaks of gastrointestinal illness are associated with multiple factors, including bacterial, viral and parasitic pathogens [3, 6, 8, 11], it is postulated that *K. septempunctata* spores are not directly linked with diarrhoea in raw fish consumers. However, the pathogenicity of *K. septempunctata* spores should be studied further in immunosuppressed individuals.

We used routine histopathology and real-time PCR to determine whether *Kudoa* spores infect adult animals, but found no evidence of *K. septempunctata* infection in any of the organs of mice orally administered various doses of *K. septempunctata* spores. Rather, spores were excreted in faeces, as evidenced by the fact that target genes were detected by qPCR analyses of stool samples from spore-administered groups after 3 hpi. Therefore, our findings suggest that adult mice are not susceptible to infection by *K. septempunctata* spores.

Taken together, our data suggest that spores of *K. septempunctata* genotype ST3 do not invade or destroy the intestinal mucosa of adult mice. Rather, these spores were excreted in faeces within a few hours following intragastric administration. This study implies that spores of *K. septempunctata* genotype ST3 (which originates from olive flounder) do not cause diarrhoea in adult mice.

## Conflict of interest

None.
